# Control of Cell Migration and Inflammatory Mediators Production by CORM-2 in Osteoarthritic Synoviocytes

**DOI:** 10.1371/journal.pone.0024591

**Published:** 2011-09-22

**Authors:** Isabel García-Arnandis, Maria Isabel Guillén, Francisco Gomar, Miguel Angel Castejón, Maria José Alcaraz

**Affiliations:** 1 Department of Pharmacology and IDM, University of Valencia, Valencia, Spain; 2 Department of Chemistry, Biochemistry and Molecular Biology, Cardenal Herrera-CEU University, Moncada, Valencia, Spain; 3 Department of Surgery, School of Medicine, University of Valencia, Valencia, Spain; 4 Department of Orthopaedic Surgery and Traumatology, De la Ribera University Hospital, Alzira, Valencia, Spain; University of Pittsburgh, United States of America

## Abstract

**Background:**

Osteoarthritis (OA) is the most widespread degenerative joint disease. Inflamed synovial cells contribute to the release of inflammatory and catabolic mediators during OA leading to destruction of articular tissues. We have shown previously that CO-releasing molecules exert anti-inflammatory effects in animal models and OA chondrocytes. We have studied the ability of CORM-2 to modify the migration of human OA synoviocytes and the production of chemokines and other mediators sustaining inflammatory and catabolic processes in the OA joint.

**Methodology/Principal Findings:**

OA synoviocytes were stimulated with interleukin(IL)-1β in the absence or presence of CORM-2. Migration assay was performed using transwell chambers. Gene expression was analyzed by quantitative PCR and protein expression by Western Blot and ELISA. CORM-2 reduced the proliferation and migration of OA synoviocytes, the expression of IL-8, CCL2, CCL20, matrix metalloproteinase(MMP)-1 and MMP-3, and the production of oxidative stress. We found that CORM-2 reduced the phosphorylation of extracellular signal-regulated kinase1/2, c-Jun N-terminal kinase1/2 and to a lesser extent p38. Our results also showed that CORM-2 significantly decreased the activation of nuclear factor-κB and activator protein-1 regulating the transcription of chemokines and MMPs in OA synoviocytes.

**Conclusion/Significance:**

A number of synoviocyte functions relevant in OA synovitis and articular degradation can be down-regulated by CORM-2. These results support the interest of this class of agents for the development of novel therapeutic strategies in inflammatory and degenerative conditions.

## Introduction

There is substantial evidence that synovitis contributes to the progression of osteoarthritis (OA). The inflamed synovium releases pro-inflammatory and catabolic mediators with ultimately destructive consequences on articular tissues. Synovial cells attach to cartilage and bone fragments and have been implicated in sustaining joint damage [1]. Pro-inflammatory cytokines produced by both chondrocytes and synoviocytes have been involved in joint destruction during OA [2], [3]. In particular, interleukin(IL)-1β activates a broad array of signaling pathways in joint tissues which shift cartilage homeostasis toward catabolism [4]. Evidence from previous studies indicate that OA synovial cells are activated by pro-inflammatory cytokines to release cytokines and chemokines, reactive oxygen species (ROS) and other mediators that likely contribute to sustain the inflammatory response [5], as well as proteases such as matrix metalloproteinases (MMPs) which act in a synergistic manner to degrade the components of connective tissue [6].

Heme oxygenase-1 (HO-1) is induced by oxidative stress and different stimuli as a cell defense mechanism due to its antioxidant and anti-inflammatory effects (reviewed in [7]). It is also known that one of the metabolites derived from HO activity, carbon monoxide (CO) elicits essential biological functions and mediates many of the effects that are attributed to HO activity. Recently, CO-releasing molecules (CO-RMs) have been synthesized as a new drug class able to reproduce many of the biological effects of HO and CO [8], [9]. We have shown previously that CORM-3 exerts anti-arthritic effects in mice [10] and CORM-2 reduces the production of some inflammatory mediators and MMPs in OA chondrocytes [11], [12]. However, evidence to show whether CO-RMs are capable of modulating the metabolism of OA synoviocytes is lacking. Therefore, we investigated the potential of CORM-2 to regulate key metabolic functions of human OA synoviocytes in relation with synovitis and joint degeneration.

## Results

### CORM-2 does not induce HO-1 in the presence of IL-1β stimulation

**Figure 1:**
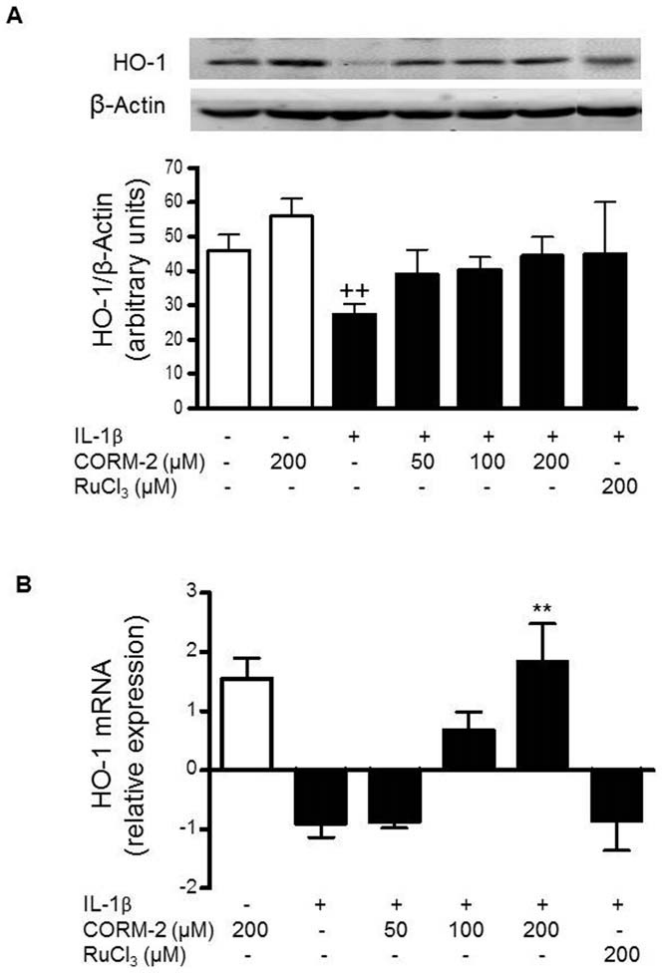
Effect of CORM-2 on HO-1 protein (A) and mRNA expression (B) in OA synoviocytes. Cells were stimulated with IL-1β (10 ng/ml) for 24 h (A) and 16 h (B) in the presence or absence of CORM-2 (50, 100, 200 µM) or RuCl_3_ (200 µM). Protein expression was determined in cell lysates by Western blotting and mRNA levels were determined by real-time PCR. Relative expression of HO-1 and β-actin protein bands was calculated after densitometric analysis. Data are expressed as mean±S.E.M. Samples from 4 patients were used. ++*P*<0.01 with respect to nonstimulated cells. **P<0.01 with respect to IL-1β.

In some cellular systems, CO-RMs have been shown to induce HO-1 [9]. To determine whether CORM-2 was able to induce HO-1 in our experimental conditions, we examined the expression of HO-1 using Western blot and quantitative PCR methods. We found that CORM-2 treatment of basal OA synoviocytes weakly increased HO-1 expression at protein and mRNA levels without reaching statistical significance (Figure 1A and 1B). As described previously [13], IL-1β decreased HO-1 expression in OA synoviocytes. In cells stimulated with IL-1β, CORM-2 increased HO-1 mRNA levels at 200 µM but failed to enhance HO-1 protein. Therefore, the possible effects of CORM-2 in OA synoviocytes stimulated by IL-1β are not dependent on HO-1 induction. As expected, no effects were obtained with the negative control RuCl_3_.

### Effects of CORM-2 on proliferation and migration of OA synoviocytes stimulated by IL-1β

**Figure 2:**
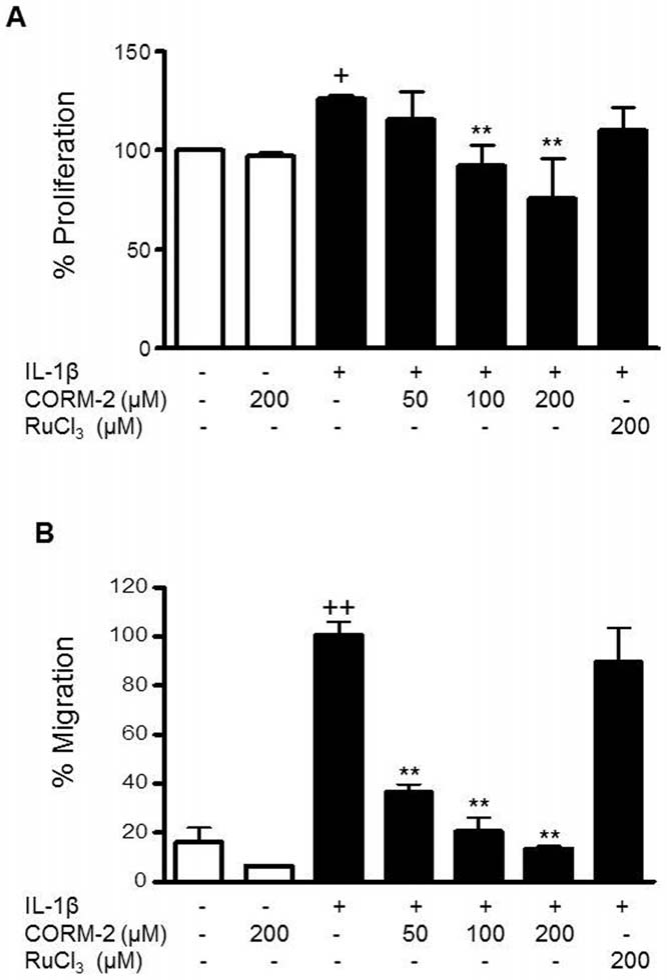
Effect of CORM-2 on synoviocyte proliferation and migration rate. Cells were stimulated with IL-1β (10 ng/ml) for 24 h in the presence or absence of CORM-2 (50, 100, 200 µM) or RuCl_3_ (200 µM). (A) Cell proliferation was determined by the MTT assay. (B) Transwell chambers were kept in culture for 24 h. Upper compartment was detached and cells migrated to the lower side were counted in 6–8 microscopic power fields. Data (A) are expressed as mean±S.E.M. of % proliferating cells, considering 100% the proliferation induced in basal conditions whereas data (B) are expressed as % of cells migrated to the lower compartment, considering 100% the migration induced by IL-1β. Duplicate samples from 6 (A) and 4 (B) patients were used. +*P*<0.05, ++*P*<0.01 with respect to nonstimulated cells. **P<0.01 with respect to IL-1β.

IL-1β activates OA synoviocytes leading to increased proliferation and migration. We investigated the possible regulatory effects of CORM-2 on these cell functions. As shown in Figure 2A, CORM-2 significantly reduced cell proliferation induced by IL-1β at the concentrations of 100 and 200 µM. Interestingly, CORM-2 strongly inhibited cell migration in a concentration-dependent manner, in the presence of IL-1β.

### Treatment with CORM-2 down-regulates chemokine production

**Figure 3:**
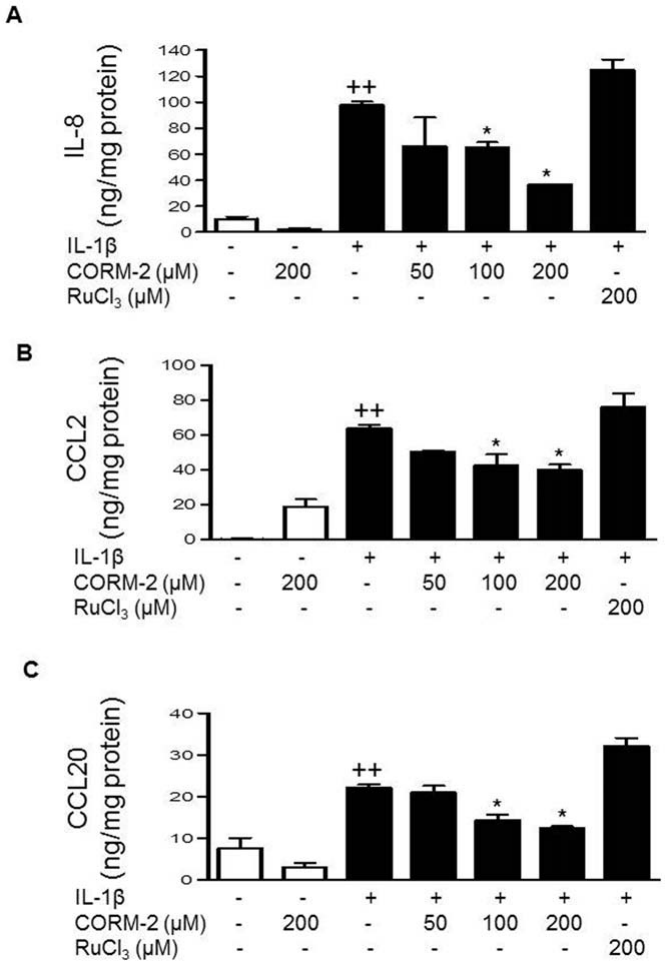
Effect of CORM-2 on the levels of chemokines released into the medium by OA synoviocytes. (A) IL-8, (B) CCL2 and (C) CCL20 protein levels. Cells were stimulated with IL-1β (10 ng/ml) for 24 h in the presence or absence of CORM-2 (50, 100, 200 µM) or RuCl_3_ (200 µM). Protein levels were determined in cell supernatants by ELISA. Data are expressed as mean±S.E.M. Duplicate samples from 6 patients were used. ++*P*<0.01 with respect to nonstimulated cells. *P<0.05 with respect to IL-1β.

**Figure 4:**
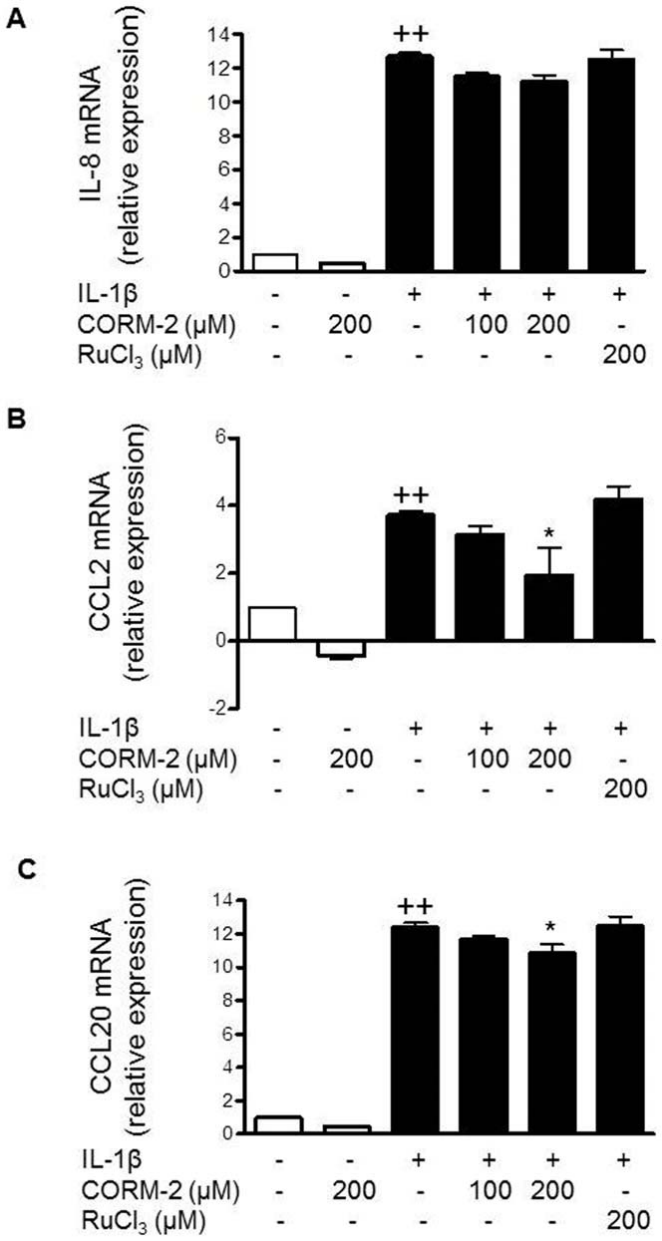
Effect of CORM-2 on chemokine mRNA levels in OA synoviocytes. (A) IL-8, (B) CCL2 and (C) CCL20 mRNA levels. Cells were stimulated with IL-1β (10 ng/ml) for 16 h in the presence or absence of CORM-2 (100, 200 µM) or RuCl_3_ (200 µM). mRNA expression was determined by real-time PCR. Data are expressed as mean±S.E.M. Duplicate samples from 3 patients were used. ++*P*<0.01 with respect to nonstimulated cells. *P<0.05, **P<0.01 with respect to IL-1β.

According to the inhibitory effects of CORM-2 in cell migration, we hypothesized that CORM-2 might affect the production of key chemokines induced by IL-1β. Therefore, we examined the presence of IL-8, CCL2 and CCL20 in the culture medium of OA synoviocytes. CORM-2 at 100 and 200 µM decreased the levels of these chemokines in the presence of IL-1β stimulation (Figure 3A-C). In addition, we assessed gene expression of IL-8, CCL2 and CCL20 in OA synoviocytes by real-time PCR. Figure 4A shows that CORM-2 did not modify IL-8 mRNA but it reduced CCL2 and CCL20 mRNA in cells stimulated by IL-1β (Figure 4B and 4C).

### CORM-2 regulates MMPs

**Figure 5:**
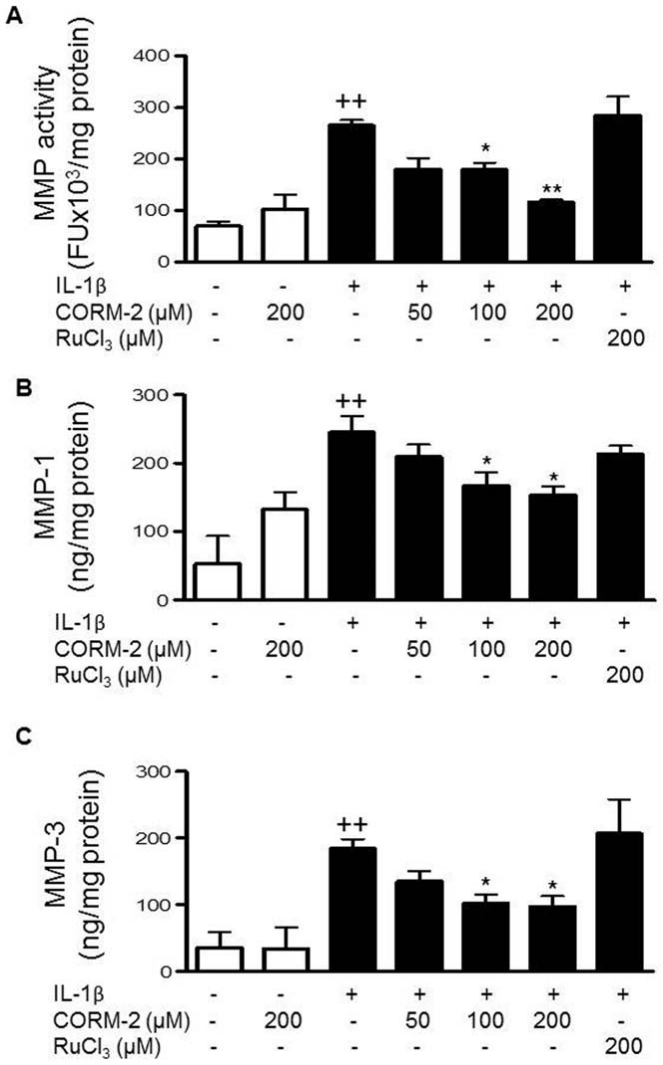
Effect of CORM-2 on MMP activity and MMP levels released into the culture medium in OA synoviocytes. (A) MMP activity, (B) MMP-1 protein, (C) MMP-3 protein levels in the culture medium. Cells were stimulated with IL-1β (10 ng/ml) for 24 h in the presence or absence of CORM-2 (50, 100, 200 µM) or RuCl_3_ (200 µM). MMP activity was analyzed by fluorometric procedures in cell supernatants (A) and protein levels were determined by ELISA in cell supernatants (B–C), Data are expressed as mean±S.E.M. Duplicate samples from 6 patients were used. ++*P*<0.01 with respect to nonstimulated cells. *P<0.05 with respect to IL-1β.

**Figure 6:**
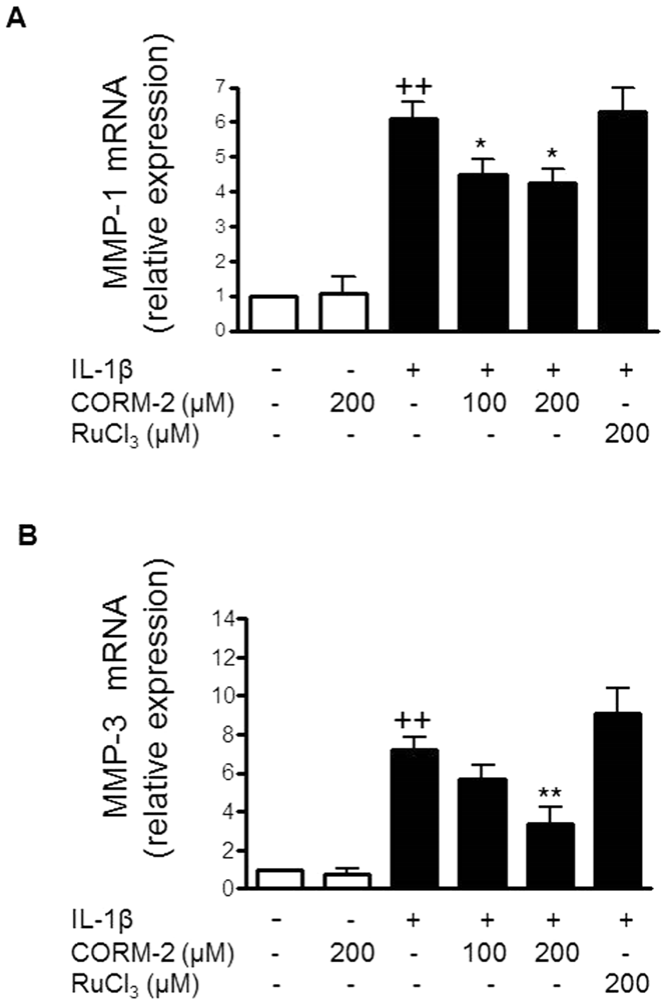
Effect of CORM-2 on MMP mRNA levels in OA synoviocytes. (A) MMP-1, (B) MMP-3 mRNA levels. Cells were stimulated with IL-1β (10 ng/ml) for 16 h in the presence or absence of CORM-2 (100, 200 µM) or RuCl_3_ (200 µM). mRNA expression was measured by real-time PCR. Data are expressed as mean±S.E.M. Duplicate samples from 6 patients were used. ++*P*<0.01 with respect to nonstimulated cells. *P<0.05, **P<0.01 with respect to IL-1β.

IL-1β stimulation of OA synoviocytes results in the induction of MMPs which play an important role in joint degradation. Figure 5A shows that MMP activity released into the medium was significantly decreased by cell treatment with either 100 or 200 µM CORM-2. In particular, MMP-1 (collagenase-1) and MMP-3 (stromelysin-1) are strongly induced by IL-1β in this cellular system and play a relevant role in OA synoviocytes [14], [15]. Our results indicated that the observed reduction in MMP activity could be dependent on inhibitory effects of CORM-2 on MMP-1 and MMP-3 secretion into the medium (Figure 5B and 5C) and mRNA expression in OA synoviocytes (Figure 6A and 6B).

### Antioxidant effects of CORM-2

**Figure 7:**
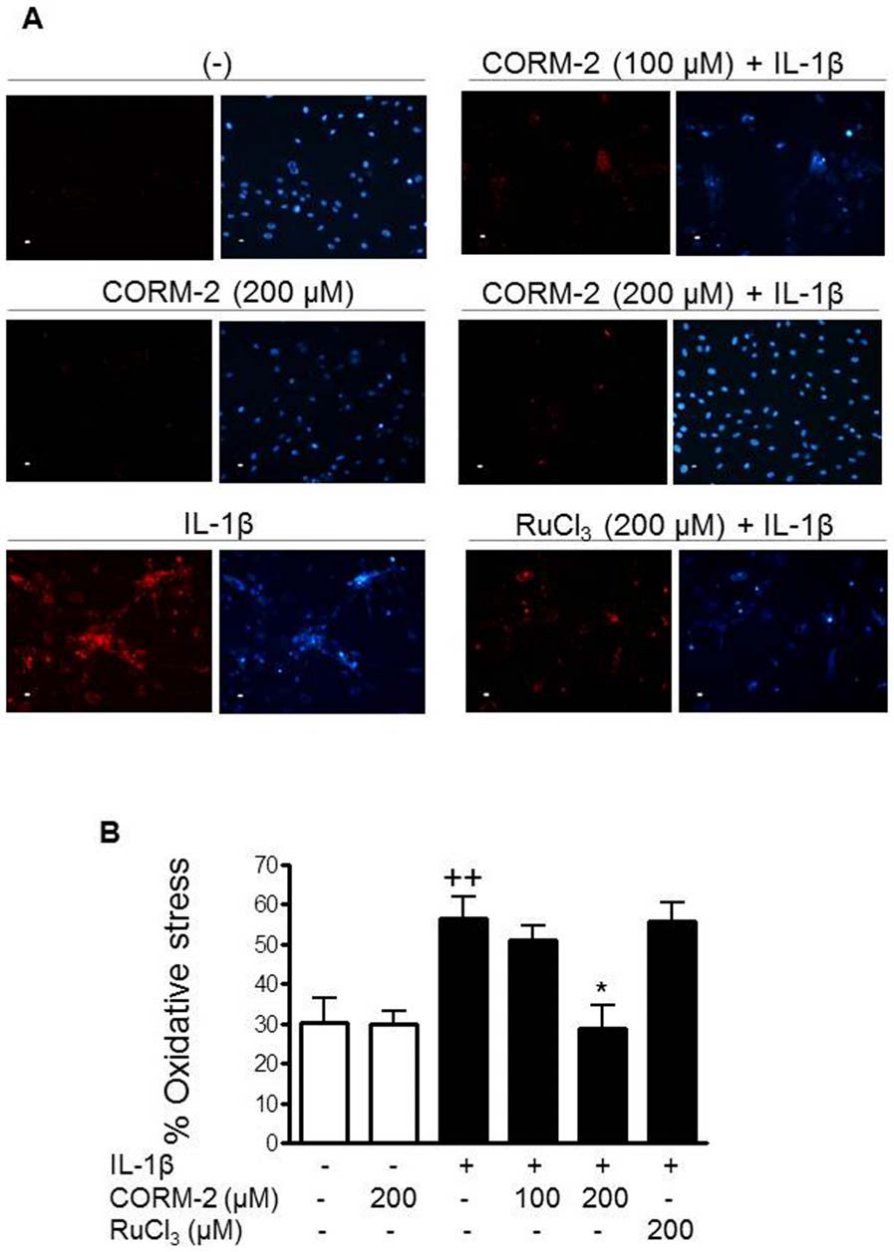
Effect of CORM-2 on oxidative stress levels in OA synoviocytes. Cells were incubated with IL-1β (10 ng/ml) in the presence or absence of CORM-2 (100, 200 µM) or RuCl_3_ (200 µM) for 30 min. Oxidative stress was measured by the oxidation of dihydrorhodamine 123. Rhodamine positive cells were counted in 6–8 microscopic power fields. Data are expressed as % of oxidative stress. Left panels: oxidative stress, right panels: DAPI. Duplicate samples from 3 patients were used. +*P*<0.05 with respect to nonstimulated cells. *P<0.05 with respect to IL-1β. Original magnification: x200.

ROS are released during the inflammatory response of joint tissues and are associated with cartilage degradation in OA [16]. It is known that products derived from HO-1 activity including CO exert antioxidant effects [7]. Therefore, we next determined whether CORM-2 could modify the production of ROS in this cellular system. Figure 7A and 7B shows that CORM-2 treatment of synovial cells had no effect on basal oxidative stress. In contrast, the increased oxidative stress produced upon IL-1β stimulation was significantly reduced by CORM-2 (200 µM).

### Signal transduction pathways involved in IL-1β stimulation of OA synoviocytes

**Figure 8:**
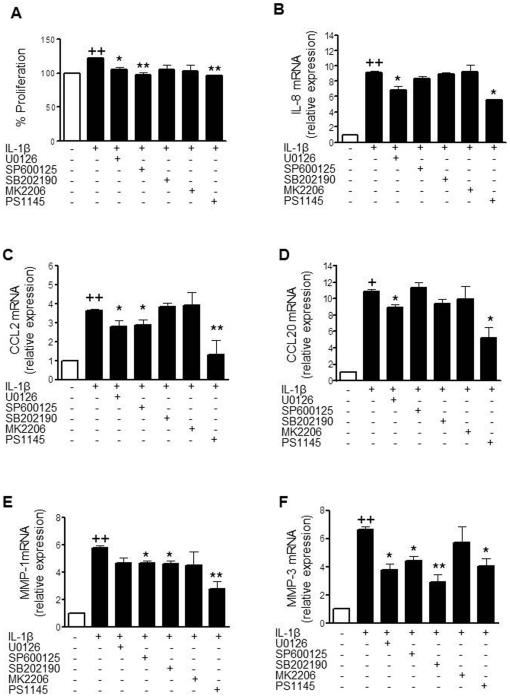
Effects of specific inhibitors of MAPK, Akt and NF-κB on IL-1β induced cell proliferation and chemokine and MMP mRNA expression in OA synoviocytes. (A) Cell proliferation, (B) IL-8, (C) CCL2, (D) CCL20, (E) MMP-1, (F) MMP-3 mRNA expression. Cells were treated with UO126 (10 µM), SP600125 (25 µM), SB202190 (10 µM), MK2206 (20 µM) or PS1145 (10 µM), specific inhibitors of MEK/ERK1/2, JNK1/2, p38, Akt and IκB kinase, respectively, 1 h before adding IL-1β for a total incubation time of 24 h (A) or 16 h (B–F). Cell proliferation was determined by the MTT method whereas mRNA expression was analyzed by real-time PCR. Data are expressed as mean±S.E.M. Duplicate samples from 3 patients were used. +*P*<0.05, ++*P*<0.01 with respect to nonstimulated cells. *P<0.05, **P<0.01 with respect to IL-1β.

To explore the potential mechanisms responsible for CORM-2 effects, we assessed the involvement of different signal transduction pathways in cell responses induced by IL-1β in our experimental conditions. For this purpose, cells were pre-treated with specific inhibitors of mitogen-activated protein kinases (MAPK) extracellular signal-regulated kinase 1/2 (ERK1/2), p38 and c-Jun N-terminal kinase 1/2 (JNK1/2) which is involved in activator protein-1 (AP-1) activation, as well as inhibitors of Akt and nuclear factor-κB (NF-κB) activation. Figure 8A shows that the proliferative effect of IL-1β was reduced when synoviocytes were treated with specific inhibitors of ERK1/2, JNK1/2 or NF-κB. Similarly, the effects of IL-1β on chemokine mRNA expression were decreased by inhibition of NF-κB and to a lesser extent of ERK1/2 for all chemokines, in addition to JNK1/2 inhibition in the case of CCL2 (Figure 8B–8D). Our results also showed that NF-κB activation is involved in MMP-1 and MMP-3 induction, with the contribution of MAPK (Figures 8E and 8F).

### Effect of CORM-2 on MAPK and Akt activation

**Figure 9:**
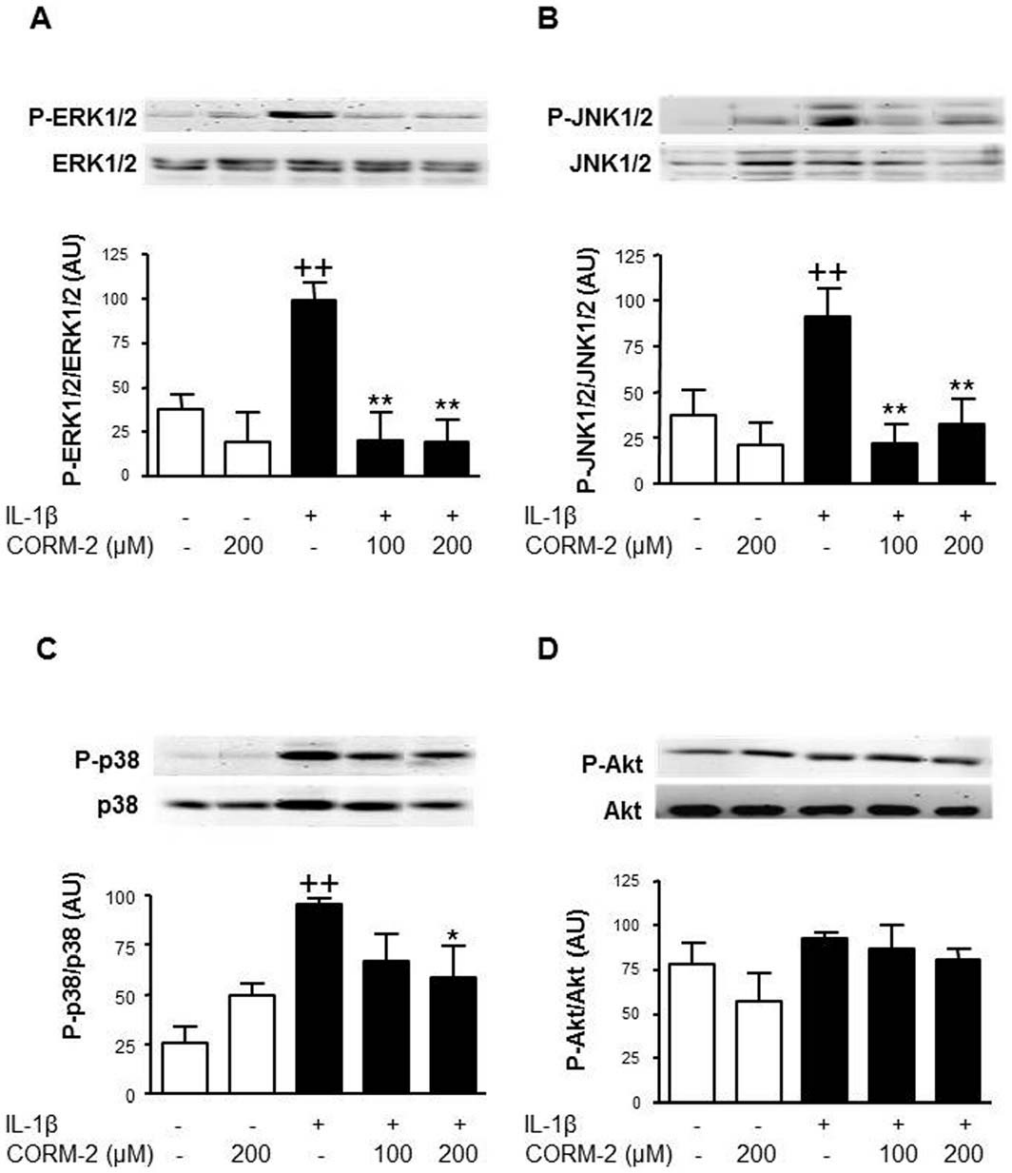
Effect of CORM-2 and IL-1β on Akt and MAPK phosphorylation in OA synoviocytes. Cells were stimulated with IL-1β (10 ng/ml) for 5 min in the presence or absence of CORM-2 (100, 200 µM). Protein expression was determined in cell lysates by Western blotting using specific antibodies against phosphorylated or total proteins. Relative expression of phosphorylated and total protein bands was calculated after densitometric analysis. AU = arbitrary units. Data are expressed as mean±S.E.M. (samples from 3 patients). ++*P*<0.01 with respect to nonstimulated cells. *P<0.05, **P<0.01 with respect to IL-1β.

Stimulation of OA synoviocytes with IL-1β quickly induces the phosphorylation of MAPK and Akt [14]. Figure 9A and 9B shows that the phosphorylation of ERK1/2 and JNK1/2 was strongly reduced by CORM-2 at 100 and 200 µM, lowering the expression of phosphorylated proteins to basal levels. In addition, p38 phosphorylation was also reduced although to a lesser extent, with a significant effect in the presence of 200 µM CORM-2 (Figure 9C). In contrast, CORM-2 treatment did not modify Akt phosphorylation (Figure 9D).

### Regulation of transcription factors activation by CORM-2

**Figure 10:**
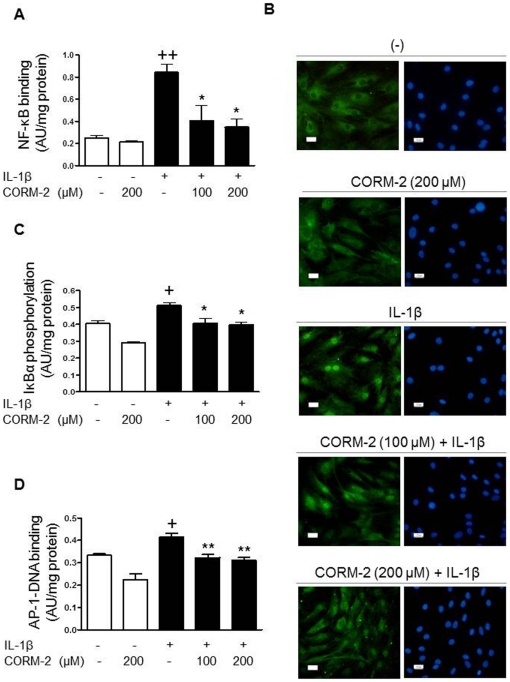
Effect of CORM-2 on NF-κB and AP-1 activation in OA synoviocytes. (A) NF-κB binding to DNA, (B) NF-κB p65 translocation (left panels: p65, right panels: DAPI), (C) IκBα phosphorylation and (D) AP-1 binding to DNA. Cells were stimulated with IL-1β (10 ng/ml) for 30 min (C) or 1 h (A, B, D) with IL-1β (10 ng/ml) in the presence or absence of CORM-2 (100, 200 µM). IκBα phosphorylation and NF-κB/AP-1 binding to DNA were determined by ELISA in cytosolic and nuclear fractions respectively, whereas p65 translocation was analyzed by immunofluorescence. Original magnification x400. Data are expressed as mean±S.E.M. of independent cultures with cells from 3 different donors. AU = arbitrary units. +*P*<0.05, ++*P*<0.01 with respect to nonstimulated cells. *P<0.05, **P<0.01 with respect to IL-1β.

Previous studies have indicated the importance of NF-κB and AP-1 activation by pro-inflammatory cytokines in the transcription of inflammatory and catabolic genes [17]-[21]. We assessed whether the observed inhibitory effects of CORM-2 on cell proliferation, and chemokine and MMP expression could be dependent on the regulation of these transcription factors. Figure 10A shows that CORM-2 did not affect basal NF-κB-DNA binding but in the presence of IL-1β stimulation, CORM-2 significantly reduced the binding of this transcription factor to DNA. We further investigated the mechanisms involved in this effect and observed that CORM-2 decreased the translocation of p65 from the cytoplasm into the nucleus (Figure 10B). As phosphorylation of NF-κB inhibitory protein (IκB) is a key step for the degradation of this protein allowing nuclear translocation of NF-κB, we also determined whether IκBα phosphorylation could be affected by CORM-2. Figure 10C shows that increased IκBα phosphorylation induced by IL-1β was significantly down-regulated by CORM-2. In addition, we studied the effects of CORM-2 on AP-1 activation. As shown in Figure 10D, CORM-2 treatment of OA synoviocytes resulted in a significant reduction in the binding of this transcription factor to DNA.

## Discussion

In the present study, we have demonstrated the inhibitory effects of CORM-2 on a number of synoviocyte functions relevant in OA. The available evidence suggests that intimal hyperplasia in OA is dependent on local proliferation of synovial lining cells and recruitment of cells of marrow origin [22]. Although the proliferative response and invasiveness of synovial fibroblasts is lower in OA compared to rheumatoid arthritis [23], pro-inflammatory cytokines activate these cells leading to enhanced proliferation, migration and production of mediators. In particular, IL-1β is a potent stimulus for cell proliferation and chemokine release in OA fibroblast synovial cells [24]. We have shown that synoviocyte proliferation induced by IL-1β was reduced in the presence of CORM-2. This response to IL-1β can be dependent on NF-κB and AP-1 activation [23], [25]. It is also known that inhibition of ERK1/2 decreases synovial fibroblasts proliferation [26]. Our results suggest that the reduced activation of these transcription factors, ERK1/2 and JNK1/2 by CORM-2 treatment could mediate the observed decrease in synovial cell proliferation.

Chemokines are important soluble factors secreted by OA chondrocytes and synoviocytes to attract and activate inflammatory cells [27]. Both cell types express chemokine receptors and are activated upon binding of specific ligands to produce inflammatory and catabolic mediators [28], [29]. In addition, synoviocytes migrate in response to chemokines. We have shown that CORM-2 treatment inhibits synoviocyte migration in the presence of IL-1β. These effects of CORM-2 on cell migration could be dependent on the inhibition of chemokine production.

Our data indicate that CORM-2 reduced the production of chemokines with an important role in synovitis. It is interesting to note that CCL2 and IL-8 produced by synovial cells have been shown to attract peripheral monocytes leading to the accumulation of macrophages in the rheumatoid synovium [30]. In addition, IL-8 and other chemokines of the same family decrease the expression of interstitial collagens type I and type III in rheumatoid synovial fibroblasts [31]. Synoviocytes from arthritic joints of mice and humans also release a large amount of CCL20, which is regulated by local production of pro-inflammatory or anti-inflammatory cytokines [32]. Interestingly, recent studies suggest that the therapeutic efficacy of anti-tumor necrosis factor-α therapies may result from the inhibition of CCL20 in rheumatoid arthritis synovium [33] as this chemokine mediates key pathogenic events such as the recruitment of Th17 cells to the inflamed joints [32].

The spontaneous production of IL-8 and CCL2 as well as the induction of the last chemokine by IL-1β are NF-κB-dependent in OA synovium [17], [18]. Our results have confirmed the participation of this transcription factor in chemokine induction by IL-1β. Nevertheless the role of NF-κB in the induction of IL-8 by IL-1β in OA synoviocytes is not completely understood as IκBα overexpression failed to modify it [34] and inhibition of NF-κB activation can give different results depending on synovial cell strains [23]. Our data show that CORM-2 exerts inhibitory effects on chemokine production induced by IL-1β in OA synoviocytes. We have also demonstrated that the down-regulation of CCL2 and CCL20 mRNA by CORM-2 could be mediated by the reduction in NF-κB transcriptional activity which would be dependent on the inhibition of IκBα phosphorylation leading to a lower rate of nuclear translocation of this transcription factor. However, CORM-2 did not modify IL-8 mRNA levels, suggesting complex regulatory mechanisms for this cytokine which would need further investigation.

Recent studies have shown that ROS may participate in the initiation and progression of OA. These mediators are released during the inflammation of the synovial membrane and can activate transcription factors such as NF-κB contributing to cartilage degradation [35]. Consistent with our previous studies in OA chondrocytes [11], we have found that CORM-2 decreases the production of ROS and NF-κB activation in OA synoviocytes stimulated with IL-1β. Our results thus suggest that the anti-inflammatory effects of CORM-2 on OA synovial cells occur, at least in part, via its ability to attenuate oxidative stress.

The irreversible destruction of articular tissues is the hallmark of both rheumatoid arthritis and OA. Collagen degradation is mediated primarily by the collagenases, MMP-1 and MMP-13, which have predominant roles in both conditions. MMP-1 efficiently degrades type I, II and III collagen and is produced mainly by synovial cells [36]. MMP-3 exhibits a broad substrate specificity and degrades gelatin, proteoglycan, fibronectin, type IV collagen, laminin, and the N propeptide of type I procollagen [37]. We have shown that CORM-2 inhibits the elevated MMP production by synovial cells activated by IL-1β. Of note, MMP-1 and MMP-3 have been known for their involvement in tissue destruction in rheumatoid arthritis and their expression in synovial fibroblast have been associated with the invasive ability of these cells [38].

The transcription factors NF-κB and AP-1 mediate MMP-1 and MMP-3 induction by IL-1β in synovial fibroblasts [19]–[21]. Therefore, our results suggest that CORM-2 reduces the production of these MMPs through the down-regulation of both transcription factors. It is known that JNK phosphorylates c-Jun and plays a key role in AP-1 activation and MMP-1 transcription in synovial fibroblasts [39]. It is also known that MAPK could cooperate with these transcription factors for efficient MMP-1 induction by IL-1β [40]. In addition, it has been reported that activation of ERK1/2 or the p38 MAP kinase pathway is sufficient to induce MMP-1 transcription in human primary fibroblasts [41]. In other articular cells, OA chondrocytes, we showed a regulatory effect of CORM-2 on ERK1/2 and p38 only [12]. In contrast, in OA synoviocytes CORM-2 strongly decreased JNK1/2 and ERK1/2 phosphorylation, with a lower effect on p38. Therefore, the inhibition of MAPK activation by CORM-2 could participate in its inhibitory effects on MMP production by OA synoviocytes.

In conclusion, our study has shown that CORM-2 down-regulates key processes contributing to synovial inflammation and articular degradation in OA. This research will help to elucidate the molecular mechanisms underlying the pharmacological effects of CO-RMs and may lead to the development of novel therapeutic strategies to prevent articular inflammatory and degenerative conditions.

## Methods

### Ethics statement

This study was approved by the Institutional Ethical Committees (*Comité Etico de Investigación en Humanos de la Comisión de Etica de Investigación Experimental de la Universidad de Valencia* y *Comité Etico de Investigación Clínica del Hospital Clínico Universitario de Valencia*) and is in compliance with all ethical standards and patients' written consent according to the Declaration of Helsinki.

### Isolation, culture and treatment of OA synoviocytes

Human OA synovial membranes were obtained from OA patients (16 females, 5 males, aged 72±4 years) undergoing total knee joint replacement surgery. Synovial specimens were finely minced and isolated by enzymatic digestion with collagenase type 1A (Sigma-Aldrich, St Louis, MO, USA) in DMEM/HAM F12 (Sigma-Aldrich) containing penicillin (100 U/ml) and streptomycin (100 mg/ml) at 37°C in 5% CO_2_ atmosphere for 16 h. The digested tissue was filtered through a 70-µm nylon mesh, washed and centrifuged. Cell viability was >95% according to the Trypan blue exclusion test. Collected cells were resuspended in DMEM/HAM F12 (Sigma-Aldrich) containing penicillin (100 U/ml) and streptomycin (100 mg/ml), supplemented with 10% fetal bovine serum (Sigma-Aldrich) and cultured at 37°C in 5% CO_2_ atmosphere until third passage (95% fibroblasts, detected by immunocytochemistry with anti-collagen I antibody, Chemicon, Millipore Iberica, Madrid, Spain) where we performed all our experiments. Synoviocytes were allowed to grow nearly until confluence and then they were incubated with CORM-2 (at different concentrations) or vehicle for 30 min before stimulation with IL-1β (10 ng/ml) (PeproTech EC, London, UK) at different times. CORM-2 (tricarbonyl dichloro ruthenium(II) dimer, Sigma-Aldrich) was dissolved in ethanol and then diluted in saline. RuCl_3_ (Sigma-Aldrich) was used as a negative control. U0126 (MEK/ERK1/2 inhibitor, 10 µM), SP600125 (JNK1/2 inhibitor, 25 µM), SB202190 (p38 inhibitor, 10 µM), MK2206 (Akt inhibitor, 20 µM) and PS1145 (IκB kinase inhibitor, 10 µM), were purchased from Sigma-Aldrich. Viability studies were performed using the Trypan blue exclusion test. None of the treatments significantly affected cell viability, which was greater than 90% in all conditions [CORM-2 (200 µM): 92.9±2.8%; IL-1β: 99.3±0.7%; CORM-2 (50 µM)+IL-1β: 91.9±2.4%; CORM-2 (100 µM)+IL-1β: 91.5+3.6%; CORM-2 (200 µM)+IL-1β: 90.5±3.4%; RuCl_3_ (200 µM)+IL-1β: 94.3±0.5%; U0126 (10 µM)+IL-1β: 96.2±7.1%; SP600125 (25 µM)+IL-1β: 96.9±9.1%; SB202190 (10 µM)+IL-1β: 92.3±1.9%; MK2206 (20 µM)+IL-1β: 94.0±1.6%; PS1145 (10 µM)+IL-1β: 91.0±2.5%].

### Determination of cell proliferation

Synoviocytes were stimulated with IL-1β (10 ng/ml) for 24 h, in the presence or absence of CORM-2. After discarding supernatants, cell proliferation was assessed by the mitochondrial-dependent reduction of 3-(4,5-dimethylthiazol-2-yl)-2,5 diphenyltetrazolium bromide (MTT) to formazan. Cells were incubated with MTT (diluted 1/10 in culture medium from a stock of 5 mg/ml in phosphate-buffered saline, PBS) during 1 h at 37°C in 5% CO_2_ atmosphere. Medium was then removed and formazan crystals were solubilized by dimethyl sulfoxide. Formazan was quantified at 490 nm.

### Chemotaxis assay

Chemotactic assay was performed in 12-well transwell plates with 8 µM pore size (BD Biosciences, Erembodegem, Belgium). Briefly, 30,000 synoviocytes/well in 200 µl DMEM/HAM F12 (Sigma-Aldrich) containing penicillin (100 U/ml) and streptomycin (100 mg/ml), and supplemented with 10% fetal bovine serum, were seeded in the upper compartment of the chamber. Supernatants harvested from cells incubated 24 h with CORM-2 in the presence or absence of IL-1β (10 ng/ml) were added to the lower side. After 24 h at 37°C in 5% CO_2_ atmosphere, upper compartment was detached. Cells migrated to the lower side of the chamber were then fixed with 4% formalin, stained with hematoxylin-eosin and counted in 6-8 microscopic power fields.

### Western blot analysis

After 24 h stimulation with IL-1β (10 ng/ml) or IL-1β+CORM-2, synoviocytes were lysed in 100 µl of buffer (1% Triton X-100, 1% deoxycholic acid, 20 mM NaCl and 25 mM Tris, pH 7.4) and centrifuged at 4°C for 10 min at 10,000 g. Protein content was determined by the DC Bio-Rad protein reagent (Richmond, CA, USA). Proteins (15–25 µg) in cell lysates were separated by 12.5% SDS-PAGE and transferred onto polyvinylidene difluoride membranes. Membranes were blocked with 3% bovine serum albumin and incubated with specific antibodies: anti-HO-1, (1∶1,000) (Stressgen, Victoria, Canada) for 2 h at room temperature, or anti-phosphorylated or total Akt, ERK1/2 or JNK1/2 (1∶500) (Cell Signalling Technology Inc., Beverly, MA, USA) and anti-phosphorylated or total p38 (1∶250) (Promega Corporation, Madison, WI, USA) overnight at 4°C. Finally, membranes were incubated with peroxidase-conjugated goat anti-rabbit IgG (DAKO, Glostrup, Denmark) and the immunoreactive bands were visualized by enhanced chemiluminescence (GE Healthcare, Barcelona, Spain) using the AutoChemi image analyzer (UVP Inc., Upland, CA, USA).

### Enzyme-linked immunosorbent assay

Synoviocytes were stimulated with IL-1β (10 ng/ml) for 24 h, in the presence or absence of CORM-2. Supernatants were harvested, centrifuged and frozen at −80°C until analysis. IL-8 and CCL2 levels were determined by specific ELISA kits from eBioscience (San Diego, CA, USA) with sensitivities of 4 and 7 pg/ml, respectively. CCL20 was determined by using a specific ELISA from R&D Biosystems (Abingdon, UK) with sensitivity of 0.47 pg/ml. MMP-1 and MMP-3 protein levels were quantified by specific ELISA kits from Raybiotech (Norcross, GA, USA) with sensitivities of 8 pg/ml and 0.3 ng/ml, respectively. IκBα phosphorylation was measured in cytosolic extracts stimulated with IL-1β (10 ng/ml) or IL-1β+CORM-2 for 30 min with K-LISA™ IKKβ Inhibitor screening kit (Calbiochem EMD Bioscience, Darmstadt, Germany). NF-κB binding to DNA was quantified by ELISA in nuclear extracts from synoviocytes stimulated with IL-1β (10 ng/ml) or IL-1β+CORM-2 for 1 h, using the Nuclear Extract Kit Active Motif for nuclei extraction followed by Trans AM™ NF-κB kit (both purchased from Active Motif Europe, Rixensart, Belgium), according to the manufacturer's recommendations.

### Real-time PCR

Following incubation for 16 h, total RNA was extracted using the TriPure reagent (Roche Applied Science, Barcelona, Spain) according to the manufacturer's instructions. Reverse transcription was accomplished on 1 µg of total RNA using random primers (TaqMan reverse transcription reagents, Applied Biosystems, Madrid, Spain). PCR assays were performed in duplicate on an iCycler Real-Time PCR Detection System using SYBR Green PCR Master Mix (Bio-Rad Laboratories, Richmond, CA, USA). Sequences of primers used have been reported previously [42]–[46]. For each sample, differences in threshold cycle (ΔCt) values were calculated by correcting the Ct of the gene of interest to the Ct of the reference gene glyceraldehide-3-phosphate dehydrogenase (GAPDH). Relative gene expression was expressed as ΔΔCt with respect to nonstimulated cells.

### Determination of MMP activity

Cells were stimulated with IL-1β (10 ng/ml) or IL-1β+CORM-2 for 24 h and supernatants were harvested, centrifuged and incubated with p-aminophenylmercuric acetate for 6 h at 37°C to activate MMPs. Aliquots of supernatants were then transferred to a 96-well plate and after addition of the 5-FAM peptide substrate (AnaSpec Inc., San Jose, CA, USA), fluorescence was measured for different times at 490 nm (excitation)/520 nm (emission) in a Victor3 microplate reader (PerkinElmer, Madrid, Spain).

### Oxidative stress quantification

Formation of intracellular ROS was detected using dihydrorhodamine 123 (Molecular Probes, Invitrogen S.A., Barcelona, Spain), which is oxidized to fluorescent rhodamine [485 nm (excitation)/534 nm (emission)]. For this purpose, synoviocytes were seeded (10,000 cells/well) in 8-well Lab-tek chambers (Nalge NuncInternational, Naperville, IL, USA) in DMEM/HAM F12 containing penicillin (100 U/ml), streptomycin (100 mg/ml) and 10% fetal bovine serum in a humidified 5% CO_2_ incubator at 37°C and kept in culture until they reach a confluence of 80% approximately. Then cells were washed with DMEM without phenol red (Sigma-Aldrich) and incubated for 15 min at 37°C with dihydrorhodamine 123 (5 µM) in DMEM without phenol red. After washing, fresh medium was added and cells were incubated with IL-1β (10 ng/ml) for 30 min in the presence or absence of CORM-2. Cell nuclei were counterstained with 4′,6-diamidino-2-phenylindole (DAPI) solution (1∶1,000). Slides were examined under a fluorescence microscope (Eclipse E800, Nikon Instruments Europe, Amstelveen, The Netherlands). Rhodamine positive cells were counted in 6–8 microscopic power fields. Each experiment was done in triplicate.

### Immunofluorescence

Synoviocytes (20,000 cells/well) were grown in 8-well chamber slides and treated with CORM-2 in the presence or absence of IL-1β for 1 h. Cells were fixed with methanol:acetic acid solution (95∶5) for 20 min at −20°C. After being washed in PBS, cells were permeabilized with 0.25% Nonidet P-40 in PBS for 10 min and incubated with rabbit polyclonal p65 antibody (Santa Cruz Biotechnologies, Santa Cruz, CA, USA) (1∶100) for 1.5 h at 37°C. Cells were then washed and incubated with secondary goat anti-rabbit antibody conjugated to Alexa Fluor® 488 (Invitrogen, Barcelona, Spain) (1∶250 in PBS) for 45 min at 37°C. Cell nuclei were counterstained with DAPI solution (1∶1,000). Slides were examined under a fluorescence microscope (Eclipse E800). Each experiment was done in triplicate.

### Statistical analysis

Results are expressed as mean±S.E.M. Statistical analysis was performed using the Kruskal-Wallis test and Dunn's post-test. *P* values<0.05 were considered significant.
